# Implementing SLMTA in the Kenya National Blood Transfusion Service: lessons learned

**DOI:** 10.4102/ajlm.v6i1.585

**Published:** 2017-04-24

**Authors:** Eric N. Wakaria, Charles O. Rombo, Margaret Oduor, Serah M. Kambale, Kimberly Tilock, Daniel Kimani, Ernest Makokha, Peter M. Mwamba, Jane Mwangi

**Affiliations:** 1 Global Communities, Nairobi, Kenya; 2 Kenya Ministry of Health, Kenya National Blood Transfusion Service, Nairobi, Kenya; 3 Division of Global HIV and TB, United States Centers for Disease Control and Prevention, Nairobi, Kenya

## Abstract

**Background:**

The Kenya National Blood Transfusion Service (KNBTS) is mandated to provide safe and sufficient blood and blood components for the country. In 2013, the KNBTS National Testing Laboratory and the six regional blood transfusion centres were enrolled in the Strengthening Laboratory Management Toward Accreditation (SLMTA) programme. The process was supported by Global Communities with funding from the United States Centers for Disease Control and Prevention.

**Methods:**

The SLMTA implementation at KNBTS followed the standard three-workshop series, on-site mentorships and audits. Baseline, midterm and exit audits were conducted at the seven facilities, using a standard checklist to measure progress. Given that SLMTA was designed for clinical and public health laboratories, key stakeholders, guided by Global Communities, tailored SLMTA materials to address blood transfusion services, and oriented trainers, auditors and mentors on the same.

**Results:**

The seven facilities moved from an average of zero stars at baseline to an average of three stars at the exit audit. The average baseline audit score was 38% (97 points), midterm 71% (183 points) and exit audit 79% (205 points). The *Occurrence Management and Process Improvement* quality system essential had the largest improvement (at 67 percentage points), from baseline to exit, whereas *Facilities and Safety* had the smallest improvement (at 31 percentage points).

**Conclusion:**

SLMTA can be an effective tool for preparing a blood transfusion service for accreditation. Key success factors included customising SLMTA to blood transfusion activities; sensitising trainers, mentors and auditors on operations of blood transfusion service; creating SLMTA champions in key departments; and integrating other blood transfusion-specific accreditation standards into SLMTA.

## Introduction

A functional blood transfusion service (BTS) is a critical component of a comprehensive healthcare system. Blood transfusion is an essential and lifesaving intervention which is key to patient treatment and management. BTS involves interrelated processes carried out by various cadres of medical and non-medical professionals. Errors may occur at any point, resulting in dire consequences for the blood donors, patients and the public at large.^1,2^

To ensure the high quality and safety of blood and blood components, a national BTS needs to have in place a comprehensive quality management system (QMS) covering the entire process from donor selection through blood donation to utilisation. This aligns with the World Health Organization’s strategy on universal access to safe blood transfusion, which promotes the safety and accessibility of blood and blood components, and the reduction of transfusion-associated risks.^3^ The strategy recommends implementation of effective quality systems for BTS, including quality management, development and implementation of quality standards, effective documentation systems, staff training and regular quality audits.^3^

The Kenya National Blood Transfusion Service (KNBTS), under the Ministry of Health, is mandated to provide safe and adequate blood and blood components in the country. KNBTS is managed through a national coordinating unit, six Regional Blood Transfusion Centres (RBTCs) and 11 satellites. The coordinating office also houses the KNBTS National Testing Laboratory (NTL), which mainly conducts confirmatory testing for transfusion transmissible infections for the RBTCs, quality assurance and reference checking. The NTL also serves as a backup testing facility for the RBTCs. The RBTC scope of service includes: (1) blood donor mobilisation, education, recruitment and retention; (2) blood collection and donor care, and laboratory testing of donated blood; (3) blood component preparation; (4) donor counselling and notification; (5) blood banking and distribution; and (6) haemovigilance. Kenya’s blood need is approximately 420 000 units per year based on the World Health Organization’s formula of 1% of the country’s total population, currently estimated at 42 million.^4^

In 2009, the World Health Organization’s Regional Office for Africa introduced the Stepwise Laboratory Quality Improvement Process Towards Accreditation (SLIPTA) and Strengthening Laboratory Management Toward Accreditation (SLMTA) programmes. SLIPTA provides a benchmark framework that measures a laboratory’s compliance with ISO 15189 on a five-star scale using a comprehensive audit tool.^5^ SLMTA, on the other hand, provides training and mentoring to support laboratories’ implementation of the QMS to achieve immediate, measurable improvement in laboratories in resource-limited settings. To evaluate progress, the SLIPTA audit tool is used before and after the SLMTA process. SLMTA has been applied to clinical and public health laboratories with great success in moving the laboratories toward accreditation and, hence, improving the quality of laboratory services.^6^ Kenya decided to apply this framework to improve quality within the BTS. While SLIPTA is based on ISO 15189 requirements and designed for clinical and public health laboratories,^7^ it is applicable to BTS given the significant laboratory aspects of blood transfusion. In addition, SLIPTA has a significant management component derived from ISO 9001, which is applicable to all organisations.

KNBTS enrolled the NTL and the six RBTCs in SLMTA in January 2013 with support from the Technical Assistance for the Implementation and Expansion of Blood Safety Program, implemented by Global Communities and funded by the US Centers for Disease Control and Prevention under the President’s Emergency Plan for AIDS Relief. This paper describes the SLMTA process at KNBTS. It shares experiences and lessons learned, and details strategies necessary to use SLMTA to promote a safe and sufficient blood supply.

## SLMTA implementation pathway in the KNBTS

### Planning

At the start of the BTS SLMTA process, Global Communities held sensitisation meetings with the NBTS management and other stakeholders to promote buy-in and share the implementation plan. The roles and responsibilities of the various players, criteria for selection of workshop participants, and mentorship approach were agreed upon.

Four champions were selected from each of the seven facilities to participate in the SLMTA workshops. These included the RBTC head, quality manager, laboratory manager and blood donor services in-charge. The selection ensured that the management, quality, laboratory (testing, component preparation, sorting and distribution) and blood donor services (collection, notification and donor care) departments were represented. In addition, the national managers in charge of administration, procurement and commodities, training and biomedical engineering from the coordination office, participated in the workshops to ensure responsibility for development and roll-out of policies, budgeting and human resource allocation, among other critical functions.

### Adaptation of SLMTA to accommodate blood safety activities

Since SLMTA was designed for clinical and public health laboratories, it had to be adapted to address most of the blood transfusion processes. While SLMTA focuses on the laboratory, BTS has processes outside the laboratory, such as blood donor recruitment, donor care, donor notification, blood component processing, blood banking and distribution to transfusing facilities. Training materials were customised to fit BTS based on available blood-specific accreditation standards, such as those from the Africa Society of Blood Transfusion. All departments of the BTS (including those outside the laboratory) were invited to the trainings. Improvement projects were selected across all of these departments.

### SLMTA implementation

**FIGURE 1:**
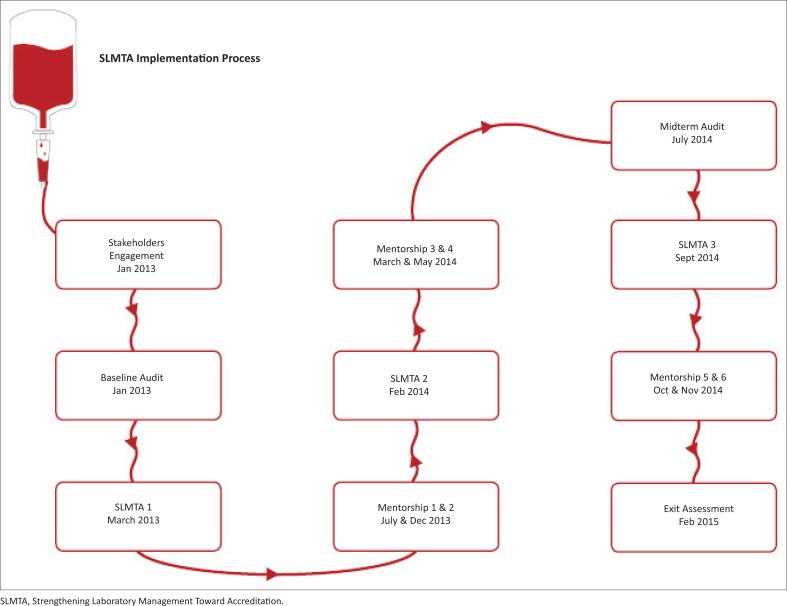
Kenya National Blood Transfusion Service SLMTA implementation process.

The standard SLMTA implementation pathway of three workshops, on-site mentorships and audits was employed.^7^ The details of the workshops, mentorships, audits and other activities are summarised in Figure 1.

Prior to the first training, the SLMTA trainers from the Kenya Medical Research Institute, a key SLMTA implementing partner in Kenya, were oriented on BTS, since most of them were drawn from clinical laboratories. The orientation resulted in customisation of the training, improvement projects and mentorship for BTS activities, as well as in facilitating content inclusion to address the implementation needs of non-laboratory staff.

### Improvement projects

After each workshop, participants implemented improvement projects related to the training content as summarised in Table 1. The improvement projects were structured to enable their implementation in various departments of KNBTS, thereby facilitating across-the-board application. The four participants trained per facility spearheaded the improvement projects implemented in their respective departments.

**TABLE 1:** List of improvement projects at the National Testing Laboratory and regional blood transfusion centres in Kenya, 2013–2015.

Workshop	Improvement projects per SLMTA curriculum	KNBTS customised improvement projects	Department responsible for implementation
Workshop 1	Monitor one of the quality indicators	Monitor units of blood collected	Blood Donor Services
		Monitor blood unit discarded	Blood Donor Services and Laboratory
		Monitor turn-around time for delivery of donor cards	Blood Donor Services
		Monitor blood component preparation	Laboratory
	Re-design laboratory layout	Re-design facility layout	All departments
	Design a competency assessment program for the laboratory and conduct some competency assessments	Design a competency assessment program for the technical staff and conduct some competency assessments	Blood Donor Services and Laboratory
	Improve workstation set-up	Improve workstation set-up	All departments
Workshop 2	Conduct a safety audit using the Safety section of the Checklist	Conduct a safety audit using the safety section of the checklist	All departments
	Introduce an inventory management system	Strengthen the KNBTS inventory management system	Central stores at coordinating office
		Strengthen inventory system at the RBTCs	RBTCs stores
	Equipment maintenance and service	Equipment maintenance and service; Procurement, maintenance, repairs, calibration and disposal	Coordinating office
		Equipment maintenance and service, preventive maintenance	All departments at the RBTCs
	Improve documentation (policies, SOPs, quality logs and checklists) in the laboratory	Develop and disseminate all documents (quality manual, policies, processes and procedures)	Coordinating office
		Disseminate the received quality and technical documents and ensure their implementation	All departments at the RBTCs
Workshop 3	Monitor internal quality control	Monitor copper sulphate solution IQC	Blood Donor Services
		Monitor TTIs, ABO grouping, † blood components IQC	Laboratory
	Monitor performance and documentation of EQA	Monitor performance and documentation of EQA	Laboratory
	Monitor specimen rejection	Monitor blood unit discard rates	Blood Donor Services
		Monitor specimen rejection	Laboratory
		Monitor blood unit expiry	Laboratory
	Monitor results of referral specimens	Monitor results of referral specimens	Laboratory
	Customer satisfaction survey	Customer satisfaction survey (focus on blood donors and transfusing hospitals)	All departments
	Conduct an internal audit using the SLIPTA checklist sections 1 to 11	Conduct an internal audit using the SLIPTA checklist sections 1 to 11	All departments

Abbreviations: EQA, external quality assessment; IQC, internal quality control; KNBTS, Kenya National Blood Transfusion Service; RBTC, regional blood transfusion centres; SLIPTA, Stepwise Laboratory Quality Improvement Process Towards Accreditation; SLMTA, Strengthening Laboratory Management Toward Accreditation; SOP, standard operating procedure; TTI, transfusion transmissible infection.

† ABO grouping refers to the classification of blood cells based on the presence or absence of the A and B antigens on the red blood cells and presence or absence of A and B antibodies in the plasma.

### Mentorship

Global Communities used a hybrid mentorship model encompassing supervision and embedment^8^ of the mentors on site for the five days following the conclusion of each workshop and for the 10 days preceding the next workshop. Mentorship methods included demonstration, side-by-side mentoring, and re-teaching of some of the aspects learnt during the workshops. Briefing meetings between the mentors and KNBTS management were held after each mentorship cycle to review progress and identify solutions to challenges faced. Furthermore, the SLMTA coordinator provided ongoing targeted mentorship that addressed emerging challenges. Mentorship was conducted by six trained mentors and the Global Communities SLMTA coordinator with each mentor assigned a KNBTS facility. The SLMTA coordinator mentored the coordinating office.

### Audits

The KNBTS facilities were audited at baseline, midterm and exit to establish status, measure progress in quality improvement, identify areas of strengths and weakness, and measure the preparedness of the facilities for accreditation. The Kenya Accreditation Service, the sole accreditation body in the country, conducted the baseline audit in January 2013 and the exit audit in February 2015. SLMTA-trained mentors conducted the midterm audit in July 2014.

The audits were based on the SLIPTA checklist, which addresses 12 quality system essentials^9^ and enables the quantification of quality improvement, allowing for determination of progress. SLIPTA assigns stars to rate the level of compliance with the ISO 15189:2007 standard, based on scores. The rating system is: zero stars (< 55%), one star (55% – 64%), two stars (65% – 74%), three stars (75% – 84%), four stars (85% – 94%), and five stars (≥ 95%).^5,7^

### Complementary activities

Global Communities facilitated additional trainings to support the accreditation process. These included internal audit, ISO 15189:2012, method validation and measurement uncertainty. The trainings were based on needs identified during audits, SLMTA workshops and mentorship sessions, and targeted staff positions that would ensure greater impact in addressing identified gaps. Gaps in blood transfusion services, such as blood donor care, were addressed by training nurses in donor care, counselling and notification. KNBTS training curriculum was used during the training in blood transfusion activities.

In addition, Global Communities supported KNBTS to develop a quality manual, safety manual, QMS standard operating procedures and technical procedures. These tools were distributed to all KNBTS facilities. Global Communities also assisted KNBTS to institute a centralised document control model to standardise documentation.

### Additional support

Global Communities supported KNBTS in other activities, including computerisation of the BTS through installation of a Blood Establishment Computer System and strengthening its financial, administrative, procurement and human resources systems. These activities were in line with SLMTA principles of quality improvement, and complemented the SLMTA activities.

Programme ownership was a key consideration; hence, SLMTA activities were included in the performance contracts of the heads of RBTCs and the quality managers. This ensured their commitment to the process and the integration of SLMTA in their day-to-day activities.

## SLIPTA audit performance

**FIGURE 2:**
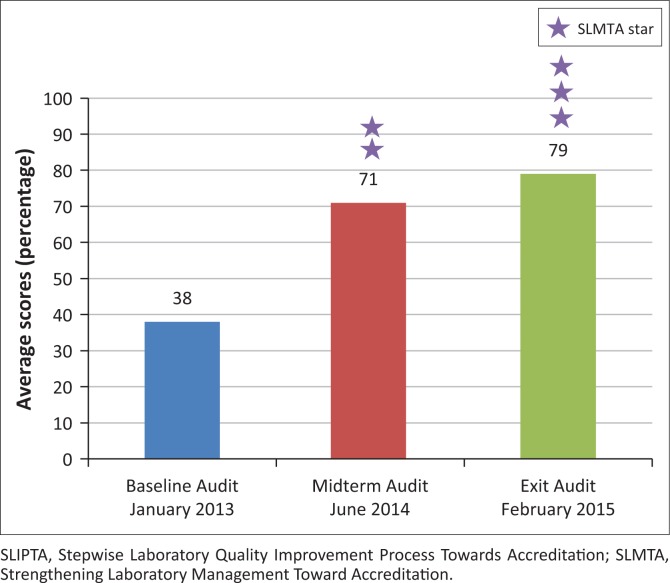
Average SLIPTA checklist scores at the SLMTA baseline, midterm and exit audits for Kenya National Blood Transfusion Service facilities. Scores were calculated from the SLIPTA checklist based on points earned for each quality system essential out of a possible total of 258. SLMTA audit stars are awarded based on the percentage of total points earned according to the following rating system: zero stars (< 55%), one star (55% – 64%), two stars (65% – 74%), three stars (75% – 84%), four stars (85% – 94%), and five stars (≥ 95%).

The maximum score possible based on the SLIPTA checklist is 258 points. Overall, the seven facilities showed steady improvement in the audit scores from an average baseline score of 38% (97 points, zero stars), to 71% (183 points, two stars) at midterm, and 79% (205 points, three stars) at exit (Figure 2). This represents an increase of 41 percentage points from baseline to exit.

**FIGURE 3:**
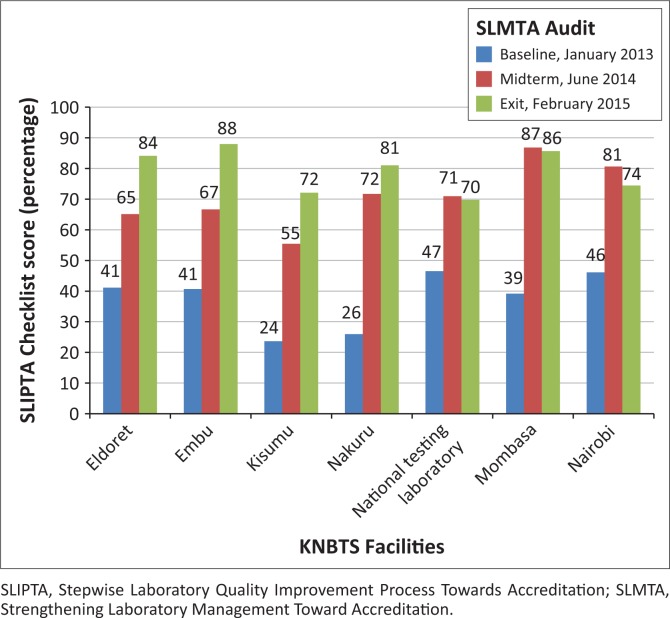
Progress of Kenya National Blood Transfusion Service facilities in the SLMTA process. For each SLMTA audit, scores were calculated from the SLIPTA checklist based on points earned for each quality system essential out of a possible total of 258.

While all of the facilities improved from baseline to exit, the level of improvement varied (Figure 3). Baseline scores ranged from 61 (24%) to 120 (47%), whereas exit scores ranged from 180 (70%) to 227 (88%). At baseline, none of the seven facilities achieved a star rating. At the midterm, one facility achieved four stars, five facilities attained two stars and one facility got one star. At the exit audit, two facilities attained four stars, two facilities had three stars and three facilities garnered two stars. One facility experienced a 7% decrease and two facilities had a 1% decrease in performance from mid-term to exit.

**FIGURE 4:**
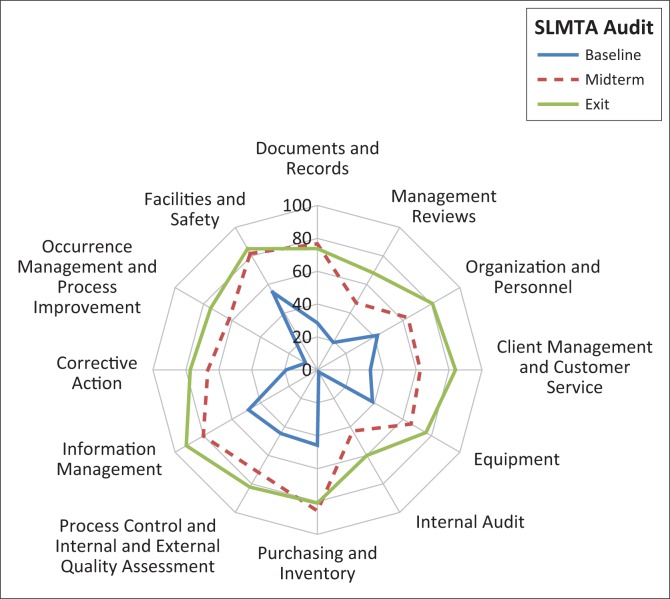
Mean quality system essential scores for Kenya National Blood Transfusion Service facilities.

Overall, improvements were observed in each of the 12 quality system essentials in the SLIPTA checklist (Figure 4). At the baseline audit, the average score for all quality system essentials was 38%, and only the score for *Facilities and Safety* was above 50%. During midterm audit, the average score for all quality system essentials increased to 71%. For *Purchasing and Inventory, Facilities and Safety* and *Information Management*, the sites registered scores of 80% and above, and the highest performance was for *Purchasing and Inventory* at 86%. However, at the midterm audits performance for *Management Reviews* (47%) and *Internal Audit* (43%) remained below 50%. At the exit audit, all seven facilities registered the highest performance in *Information Management* (92%), *Facilities and Safety* (85%) and *Client Management and Customer Service* (84%). Comparing the baseline and exit audits, marked percentage point improvements were observed for *Occurrence Management and Process Improvement* (67 percentage points), *Internal Audit* (59 percentage points) and *Corrective Action* (58 percentage points). Areas with the least improvement across the BTS facilities were *Facilities and Safety* (31 percentage points), *Purchasing and Inventory* (38 percentage points), and *Equipment* (38 percentage points).

The SLMTA process resulted in substantial quality improvements in the KNBTS. The seven facilities, which started with an average score of 38% at baseline, improved to 79% at the exit assessment, registering an increase of 41 percentage points within two years. By comparison, results from 302 laboratories participating in the SLMTA programme globally showed a 25 percentage point increase from baseline to exit audit over an average of 16 months.^6^ Clinical laboratories enrolled in the SLMTA programme in Kenya generally realise a three-star rating within nine months but stagnate at three stars; that is, the majority are unable to attain a higher star rating.^10^

Several factors contributed to the successful application of SLMTA in the KNBTS. Identifying participants from key departments in BTS to champion SLMTA ensured across-the-board quality improvement. This approach also ensured that there was buy-in and that all staff were involved in the SLMTA process in line with the World Health Organization’s integrated strategy to promote the safety and accessibility of blood and reduce the risks associated with transfusion.^3^ Cross-cutting staff involvement also ensured that the SLMTA process was embraced across the facilities and helped to overcome the notion that SLMTA was only for the laboratory department. In some departments, the champions created quality teams to oversee the improvements. Both ISO 15189 and ISO 9001 recognise the involvement of people as one principle of quality management.^11^ Our experience suggests that the impact of a QMS is greatest when managers draw on the participation, experience, and knowledge of the entire staff. This created ownership of the QMS in the KNBTS.

Improvement projects are a critical pillar of the SLMTA programme. For BTS, the improvement projects were contextualised to address the interlinked processes involved in blood transfusion. As such, the improvement projects were segmented and customised per department. Segmentation ensured that the improvement projects were relevant, focused on measurable changes and used department-derived data to stimulate departmental changes. This approach not only ensured institutionalisation of quality improvements but also increased the capacity and efficiency of service delivery. For example, the improvement projects for the blood donor services department focused on increasing blood collection, improving the turn-around time for delivery of donor cards, reducing the number of unsafe donors (and subsequently blood discard rates), and increasing donor notification to meet set standards. SLMTA learning activities were also adapted to blood transfusion. SLMTA has 44 training activities that need to be covered in approximately 60 hours spread over three workshops.^7^ As some of the SLMTA learning activities were not wholly applicable to BTS, BTS-specific learning activities were developed using the same principles used to derive SLMTA learning activities. For example, process mapping in the cross-cutting module was modified to include mapping of blood donor recruitment and mobilisation and blood issuance and distribution, as opposed to mapping the process from the ordering of a test as per processes in clinical laboratories.

Despite these improvements, challenges were observed, some of which were facility-specific, whereas others were cross-cutting. The six RBTCs share the same QMS, and have similar management structures, test menus and staffing. Therefore, it would be expected that performance would be similar among them; however, this was not the case. The facilities that scored the highest demonstrated teamwork and greater ownership of the SLMTA programme; involvement of all staff in activities led to institutionalisation of SLMTA and integration into day-to-day activities. Unlike lower-scoring facilities, these facilities had regular staff meetings, frequent internal audits and structured management reviews, demonstrating greater understanding and application of a QMS. Ownership and teamwork also resulted in innovations to address identified nonconformities. One of the facilities with four stars at the exit audit had experienced a recurring non-conformity in equipment maintenance. They addressed it by negotiating a service agreement with the biomedical engineer of the neighbouring hospital to service the equipment. Another facility that achieved four stars instituted an internal quality control programme for blood components by agreeing with a neighbouring county’s referral hospital to use their haematology analyser to perform quality checks for selected blood components.

The NTL, which performs routine and confirmatory testing for transfusion-transmissible infections for the satellites and occasionally for the RBTCS, was among the lowest-star-rated facilities, despite its proximity to the head office and its limited scope of service. Several factors may have contributed to this suboptimal performance, key among these being that the laboratory was assessed as part of the coordinating office and the non-conformities of the coordinating office were thus reflected in its audit. In addition, the laboratory occasionally experiences a heavy workload, so staff prioritise the workload instead of quality improvement activities. A similar pattern of performance was noted for the Rwanda National Reference Laboratory.^12^

Across the board, the KNBTS facilities experienced challenges in achieving improvement in some of the quality system essentials. It was observed that the least-improved quality system essentials – *Facilities and Safety, Purchasing and Inventory, Equipment*, and *Documents and Records* – all require substantial involvement of the coordinating office and therefore any failures at that office affects all facilities. Common non-conformities observed in these quality system essentials included lack of monitoring of supplier performance, frequent depletion or lack of supplies, lack of equipment calibration and service, weakness in document control, failure to perform method verification, and a deficient fire-safety programme. These issues require resources and commitment from the senior management of KNBTS. KNBTS, like many institutions implementing SLMTA in several countries, relies heavily on funding from the US President’s Emergency Plan for AIDS Relief for its activities.^13^

## Lessons learned

While the initial focus of SLMTA was on clinical and public health laboratories, our results indicate that it can be used effectively for BTS with some adjustments to the implementation approach. The trainers, mentors and auditors need to be oriented on BTS and activities to make them more effective in the application of SLMTA in BTS. For SLMTA to be effective and sustainable within BTS, all cadres and departments of the BTS should be engaged. The programme built in-house capacity by training four quality managers as SLMTA trainers.

Referring to other blood transfusion-specific accreditation standards during customisation of the training materials ensured that areas such as donor management were appropriately addressed. The Africa Society for Blood Transfusion stepwise accreditation programme^14^ was a key reference standard during the customisation.

Coordination of activities was enhanced by joint planning among all stakeholders. This not only ensured that agreed-upon timelines were observed, it also resulted in the leveraging of resources from various partners to support KNBTS.

### Limitations

This is a descriptive study based on programmatic activities and as such is subject to limitations as described below. With only seven facilities under observation, the sample size was small. However, KNBTS has only seven facilities, and all seven implemented SLMTA and were included in the study. Auditing the NTL as part of the national office resulted in the non-conformities of the national office being attributed to and reflected on the scores of the NTL, which may not have reflected the true performance of the NTL. The exit audit was conducted four months after workshop three; this period included the Christmas season and as such, KNTBS facilities did not have sufficient time to fully implement the lessons learned in the final workshop. The exit audit was conducted so soon after the workshop due to a change in the grant cycle by the donor, which resulted in reduction of the programme’s operating period by four months and subsequent close out of technical assistance support.

### Conclusion

SLMTA can be used to measurably improve QMS in BTS facilities and effectively prepare them for ISO accreditation. The KNBTS successfully used SLMTA to advance toward accreditation, progressing from zero stars to an average of three stars at the exit audit within a period of 24 months. Innovative approaches, including customising SLMTA to BTS activities; sensitising trainers, mentors and auditors on BTS and appointing SLMTA champions in key departments, contributed to the successful application of SLMTA within the KNBTS.

With the realignment in the President’s Emergency Plan for AIDS Relief to focus more exclusively on HIV care and treatment, funding for blood safety has decreased significantly. KNBTS needs to identify sustainable funding sources to address these issues, sustain the gains achieved and apply for accreditation. SLMTA remains a practical and effective approach for improving quality systems with BTS and should be considered in resource-constrained settings.

**Table:** 

Lessons learned
To effectively implement SLMTA in blood transfusion services, the trainers, mentors and auditors needed to be oriented on blood transfusion service activities. All cadres and departments of the blood transfusion service were engaged in the process by ensuring the improvement projects were segmented and customised per department. Customization of SLMTA training materials based on available blood-specific accreditation standards was necessary to ensure adaptation of SLMTA to needs of blood transfusion services. Additional support and other SLMTA-complementary activities were critical in ensuring quality improvement in the blood transfusion service. Joint planning among all stakeholders was key in ensuring coordination of activities.
